# Dutch GPs’ experience of burden by euthanasia requests from people with dementia: a quantitative survey

**DOI:** 10.3399/bjgpopen20X101123

**Published:** 2020-12-09

**Authors:** Jaap Schuurmans, Chantalle Crol, Marcel Olde Rikkert, Yvonne Engels

**Affiliations:** 1 GP, Radboud University, Groesbeek, The Netherlands; 2 Researcher, Department of Anesthesiology, Pain and Palliative Medicine, Radboud University, Nijmegen, Netherlands; 3 Primary Care Physician of the Elderly, Department of Anesthesiology, Pain and Palliative Medicine, Radboudumc, Nijmegen, The Netherlands; 4 Professor, Department Geriatric Medicine, Radboud University, Nijmegen, The Netherlands; 5 Professor in Meaningful Healthcare, Anesthesiology, Radboudumc, Nijmegen, The Netherlands

**Keywords:** euthanasia, dementia, general practitioners, support, burden

## Abstract

**Background:**

In the Netherlands during the past decade, a growing number of people with dementia requested euthanasia, and each year more of such requests were granted.

**Aim:**

To obtain quantitative insights into the problems and needs of GPs when confronted with a euthanasia request by a person with dementia.

**Design & setting:**

A concept survey was composed for GPs in the Netherlands. Expert validity of the survey was achieved through pilot testing.

**Method:**

A postal survey was sent to a random sample of 900 Dutch GPs, regardless of their opinion on, or practical experience with, euthanasia. Collected data were analysed with descriptive statistics.

**Results:**

Of 894 GPs, 423 (47.3%) completed the survey, of whom 176 (41.6%) had experience with euthanasia requests from people with dementia. Emotional burden was reported most frequently (*n* = 86; 52.8%), as well as feeling uncertain about the mental competence of the person with dementia (*n* = 77; 47.2%), pressure by relatives (*n* = 70; 42.9%) or the person with dementia (*n* = 56; 34.4%), and uncertainty about handling advance euthanasia directives (AEDs) (*n* = 43; 26.4%). GPs would appreciate more support from the following: a support and consultation in euthanasia in the Netherlands (SCEN) physician (an independent physician for support, information, and formal consultation around euthanasia) (*n* = 291; 68. 8%); a geriatric consultation team (*n* = 185; 43.7%); the end-of-life clinic (*n* = 184; 43.5%); or a palliative care consultation team (*n* = 179; 42.3%). Surprisingly the need for moral deliberation was hardly mentioned.

**Conclusion:**

The reported burden and the rise in numbers and complexity of euthanasia requests from people with dementia warrants primary care support. There needs to be easier access to colleagues with expertise, and training on end-of-life care needs of patients with dementia and their caregivers.

## How this fits in

There has been a gradual increase in legalisation on euthanasia in countries all over the world. The Netherlands was one of the first countries that legalised euthanasia in 2002. A growing number of people with dementia request euthanasia, and growing numbers are receiving it. Mostly, GPs are confronted with such requests, and a previous qualitative study showed that dealing with such requests is burdensome. This study provides quantitative insights into GPs’ burden and the need for support when dealing with euthanasia requests from people with dementia.

## Background

Euthanasia and physician-assisted suicide have been legalised in a growing number of countries; although, the practices are still only legal in a small number of countries. In all countries where it is legalised, euthanasia primarily concerns patients with cancer. Existing data do not indicate widespread abuse of these practices,^1^ but there is much debate concerning performing euthanasia, physician-assisted suicide, or other life-ending procedures in relation to vulnerable patients.^2^ Since 2002, euthanasia has, under strict conditions, been regulated by the Dutch law, as stated in Article 2 of The Termination of Life on Request and Assisted Suicide Act.^3^ At the first stage of its implementation, most requests and performances concerned terminal patients with cancer.^4^ However, during the past decade the number of euthanasia cases in people with dementia has increased sixfold, from 25 (of 3136 cases in total) in 2010 to 146 cases (of 6125 in total) in 2018.^4^ As most people with dementia, especially in the early stages of the disease, live at their own home,^5^ GPs in particular are confronted with euthanasia requests.^4^ A recent interview study showed that these requests and procedures are burdensome for GPs; for example, they experience pressure from relatives, have problems judging the person with dementia's mental capacity, and have to deal with Dutch society’s stigmatisation of dementia.^6^

Indeed, Dutch society considers dementia a 'horrible' disease and synonymous with unbearable suffering, which is sustained by the growing media attention.^7–9^ Although most people with dementia live at home and will never reach an advanced stage of the disease, many people expect a catastrophic disease course, and fear ending up in a nursing home without any quality of life. Consequently, a growing number of people in the Netherlands draw up an advance euthanasia directive (AED) and share it with their GP.^10^ Although an increasing number of countries have legalised euthanasia,^1^ only in the Netherlands an AED can replace a verbal request for euthanasia in a later stage of dementia, if all other due care criteria are met.^11^ Despite the options given, dealing with AEDs from people with dementia appeared burdensome for GPs.^6^ Not having the same expectations as, and disagreeing with, relatives about AEDs, and the timing of euthanasia contributes to this burden.^12,13^ (Re)discovering the right balance between the physician’s professional responsibility and the patient’s and relatives’ autonomy in such cases has been recommended.^14^

Recently, a Dutch case was evaluated against criminal law that raised GPs’ concerns even more around euthanasia in people with dementia.^15^ Finally, a court in The Hague determined that the woman in question with advanced dementia who was given euthanasia, and whose AED adequately represented her wishes, received legally and professionally sound care. The case against the physician, accused of murder, was dismissed.^16^ This first-ever euthanasia court case is seen as threatening in primary care across the Netherlands, as GPs typically carry out 85% of all euthanasia cases.^17^ This case demonstrates the challenges and ethical concerns GPs face when dealing with euthanasia requests and AEDs from people with dementia. Therefore, the study aimed to answer the following research question: what are the experienced burden and support needs of Dutch GPs when confronted with a euthanasia request by a person with dementia?

## Method

### Study design and participants

A quantitative survey was performed in January 2019. The addresses of a representative sample of 900 Dutch GPs were received from a Dutch institute for healthcare research. GPs with or without experience with euthanasia requests, or euthanasia performance in general, or with people with dementia specifically, were invited to take part, regardless of their opinion about euthanasia. Exclusion criteria were being retired or no longer working as a GP.

### Survey

Since no validated questionnaire to answer the research question was available, and no comparable study had been performed before, a survey was developed (Supplementary Appendix 1). Based on a literature search, a qualitative interview study,^6^ and two expert meetings,^18^ a concept survey was composed. Expert validity of the survey was achieved through pilot testing by six GPs, an ethicist, a journalist, a geriatrician, and an older persons' psychiatrist, and adapted where necessary.

The survey took 15 minutes to complete. Response options included ‘yes’ or ‘no’ or multiple-choice options, with free-text room available when participants selected the option 'other'.

The survey started with questions characterising personal and clinical practice demographics. Next, questions followed that were focused on GPs’ experiences with AEDs, with euthanasia requests and euthanasia performance in general, and regarding people with dementia. Experienced burden with regard to euthanasia requests or performance was explored with eight multiple-choice items (emotional burden; pressure from respectively the patient; emotional pressure from relatives; uncertainty about the technical performance; uncertainty about the mental competence of the patient; uncertainty about the AED; time pressure; and no burden).

To gain insights into the support needs of the past and support wishes for the future, eight multiple-choice options could be chosen: consultation of, respectively, a palliative care expert; a geriatric consultation team; a spiritual care provider; the expert centre for euthanasia; a PaTz-group (a group of GPs and district nurses who meet six times a year under the supervision of a palliative care consultant to identify early their patients who need palliative care); a SCEN physician (for support, information, and formal consultation around euthanasia); moral deliberation; and no support needs.

To explore wishes for training to increase knowledge and skills around dementia, eight multiple-choice options were provided: communicating end-of-life aspects; signalling symptoms in cognitively restricted people; dealing with pressure from relatives; legislation and its interpretation regarding euthanasia for this patient group; advance care planning; the dementia disease trajectory; AEDs; and no wishes to increase knowledge or skills.

Finally, it was asked whether recent discussions around euthanasia in people with dementia influenced the GP’s own practice with five multiple-choice options: yes, more reserved; yes, more fearful for the legal process; yes, more likely to forward such a patient to a colleague or the expert centre for euthanasia; yes, more often consulting other healthcare professionals; and no influence.

### Procedure

A code list was generated for the unique codes of the surveys and names of the GPs. The survey, for each GP with a unique code, an information letter, and a self-addressed return envelope were sent in January 2019 to the GPs by mail. Non-responders received a reminder 3 weeks later. Participation in this study was voluntary and data were processed anonymously.

Data of the completed surveys were entered in Castor, a valid database.

### Statistical analysis

All data were analysed using SPSS software (version 25). Frequencies with percentages and means with standard deviations (SDs) were used to describe the characteristics. To study differences in experienced burden of GPs between euthanasia and euthanasia requests of other patient groups and of people with dementia, χ^2^ were performed.

## Results

### Recruitment

**Figure 1.**
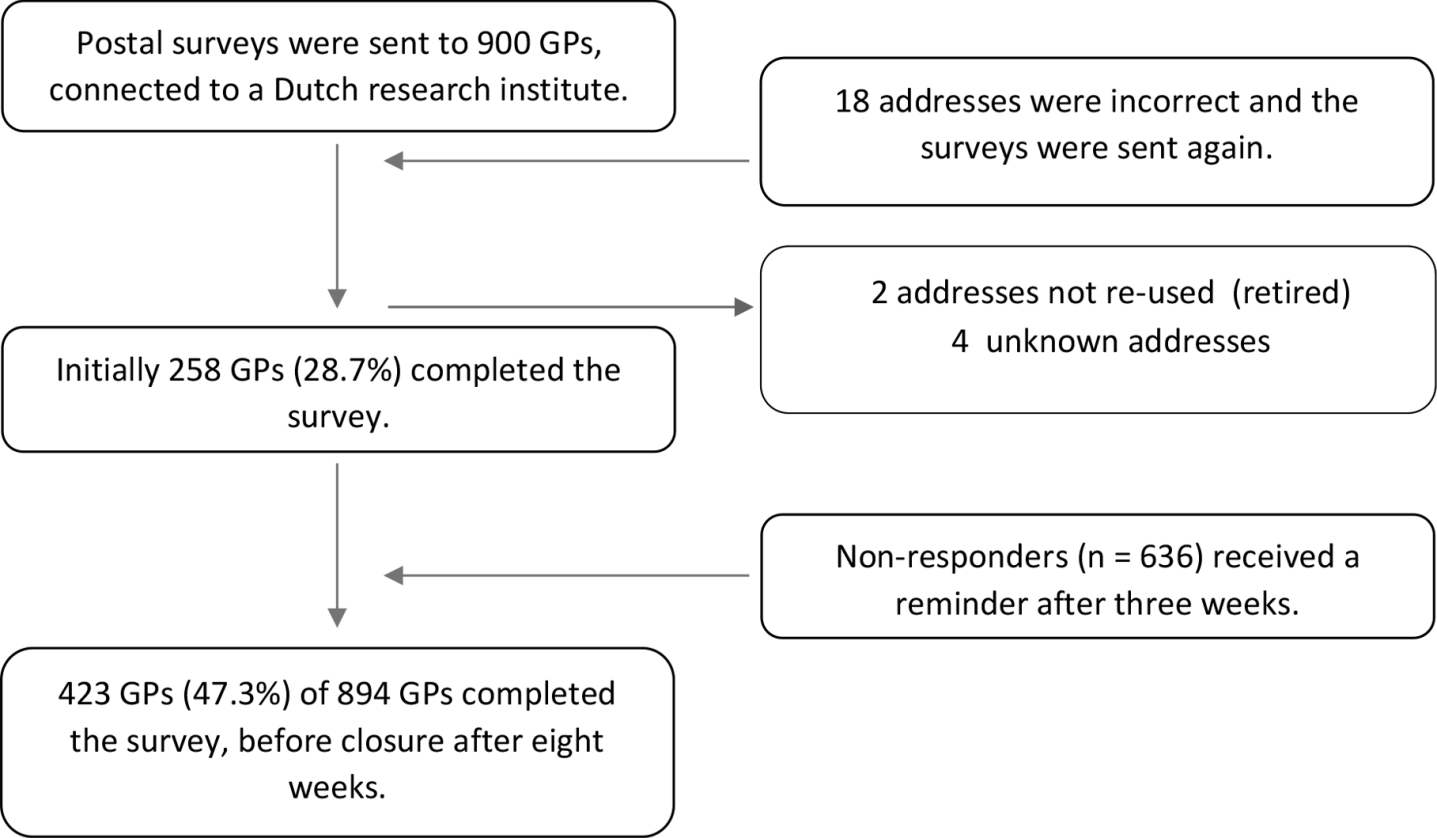
Study flow diagram.

Of the 894 included Dutch GPs, 423 (47.3%) completed the survey before closure after 8 weeks. Figure 1 is the study flow diagram, describing the procedure and response rate initially and after a reminder.

### Characteristics and experience with euthanasia and AEDs

There was an equal division between males and females, and the majority of the GPs worked as a regular in a general practice. The mean age was 48 years with a mean of 17 years’ experience (Table 1).

**Table 1.** GPs’ characteristics (*n* = 423)

Characteristics	
Mean age, years (SD)	48.1 (9.8)
Mean experience as GP, years (SD)	16.5 (9.4)
	***n*** **(%)** ^a^
Sex	
Male	207 (49.2)
Female	214 (50.8)
Kind of GP	
Regular	390 (92.6)
Locum	30 (7.1)
Having had at least one euthanasia request from a patient with another disease than dementia	410 (96.9)
Having at least once performed euthanasia in a patient with another disease than dementia	340 (86.5)^a^
Having had at least one euthanasia request from a patient with dementia	
Yes, competent	135 (32.9)
Yes, incompetent	41 (10.0)
No	249 (60.7)^a^
Having at least once performed euthanasia in a person with dementia	
Yes, competent	37 (8.7)
Yes, incompetent	3 (0.7)
No	384 (90.7)
Having no experience with euthanasia in people with dementia, but possibility of performing it in future	
Yes	173 (45.1)
No, but will refer to a colleague	180 (47.9)
No, and will not refer to a colleague	31 (7.0)
Estimated number of AEDs received per month	
<1	195 (46.7)
≥1	228 (53.9)
Estimated percentage of AEDs containing euthanasia requests in case of dementia ≥50%	222 (52.6)^a^

SD = standard deviation; AED = advanced euthanasia directive. ^a^Number of missing variables among 2–17 GPs.

Of the responding 423 GPs, 340 (80.4%) had at least once performed euthanasia. Two out of five (*n* = 176; 41.6%) had at least once received a request for euthanasia from a person with dementia. Of those 176 GPs, 40 in total also had performed euthanasia in a person with dementia (22.7%: 37 on patients judged competent for this decision and three judged incompetent).

Of the 384 GPs who had never performed euthanasia in a person with dementia, 173 (45.1%) could imagine performing euthanasia in such a patient in the future. An almost equal number of 180 GPs (47.9%) would always refer such a patient to a colleague or the Dutch expert centre for euthanasia. Only a small number of GPs (*n* = 31, 7.0%) were convinced that they would never perform euthanasia in people with dementia and also never refer a patient with such a request to a colleague.

Over half of the GPs (*n* = 228; 53.9%) estimated that they received one or more AEDs per month, and that more than half contained preferences around euthanasia in cases of dementia.

### Burden

Regarding euthanasia requests or procedures for people with a disease other than dementia, the majority of the GPs experienced emotional burden (284; 69.0% versus 86; 52.8%; *P* = 0.000), or uncertainty around the technical performance (107; 26.1% versus 12; 7.4%; *P* = 0.000) (Table 2). When it concerned a person with dementia, many more GPs experienced uncertainty about the mental competence of the patient (77; 47.2% versus 51; 12.4%; *P* = 0.015 in the other patients), and about dealing with AED (26.4% versus 4.1%; 0.046). Pressure from patients (respectively 155; 37.8% and 56; 34.4%; *P* = 0.167) or relatives (respectively 42.2% and 42.9%; *P* = 0.560) and time pressure (respectively 167; 40.7% and 16; 9.8%; *P* = 0.121) did not significantly differ between patients with and without dementia.

**Table 2.** Burden experienced by GPs in euthanasia practice for people with dementia and other diseases (several answers possible)

Burden experience	Patients with another disease than dementia *n* (%)^a^	Patient with dementia *n* (%)^b^	χ^2^ ^a^
Emotional burden GP	284 (69.0)	86 (52.8)	0.000
Pressure from the patient	155 (37.8)	56 (34.4)	0.167
Pressure from relatives	173 (42.2)	70 (42.9)	0.560
Uncertainty technical performance	107 (26.1)	12 (7.4)	0.000
Uncertainty mental competence	51 (12.4)	77 (47.2)	0.015
Uncertainty AED	17 (4.1)	43 (26.4)	0.046
Time pressure	167 (40.7)	16 (9.8)	0.121
No burden experience	22 (5.4)	14 (8.6)	0.001

AED = advanced euthanasia directive. ^a^ *n* = 410 (experiences with request or performance). ^b^ *n* = 163; 14 GPs did not respond to this question.

A third of the GPs declared that they had become more reserved with performing euthanasia in people with dementia in the light of a recent public debate related to the court case (Table 3).^15^ One out of four GPs (*n* = 102; 24.2%) had become more fearful for the legal processes, 82 (19.4%) were more likely to refer these patients to a colleague or the expert centre for euthanasia, and 89 (21.1%) intended to consult other healthcare professionals more often. One in three GPs (*n* = 153; 36.3%) stated they were not influenced by the media attention.

**Table 3.** The impact of a 2019 debate on euthanasia in people with dementia on GPs future behaviour in questions for euthanasia in people with dementia (*n* = 422)

Yes, I am more reserved in performing euthanasia	132 (31.3)
Yes, I am more fearful for the legal processes	102 (24.2)
Yes, I am more likely to forward these patients to a colleague or end-of-life clinic	82 (19.4)
Yes, I consult other healthcare professionals more often	89 (21.1)
No, no influence	153 (36.3)

### Support needs

The anticipated need for support in future euthanasia procedures with people with dementia appeared much higher than support asked in the past (Table 4). About half of the GPs who had experience with such procedures, had consulted a SCEN physician in earlier trajectories (*n* = 83; 50.9%), and even more responders would prefer this in the future (*n* = 291; 68.8%). Furthermore, support of a geriatric consultation team (*n* = 185; 43.7%), a palliative care consultation team (*n* = 179; 42.3%) or the expert centre for euthanasia (*n* = 184; 43.5%) were most often mentioned as support sources needed. Support of a spiritual care provider had rarely been asked in the past (*n* = 2; 1.2%) and hardly mentioned as needed in the future (*n* = 16; 3.8%). Hardly any GPs (*n* = 2; 1.2%) had experience with a moral deliberation around such cases, and only a minority (*n* = 30; 7.1%) expected to need this kind of support in the future.

**Table 4.** Support asked by GPs in the past and needed in the future by other healthcare professionals with regards to euthanasia procedures for people with dementia

	Support asked in the past	Support needs future
	*n* (%)^a^	*n* (%)^b^
Consult palliative care	34 (20.9)	179 (42.3)
Geriatric consult team	38 (23.3)	185 (43.7)
Spiritual care provider	2 (1.2)	16 (3.8)
Expert centre for euthanasia	52 (31.9)	184 (43.5)
Moral deliberation	2 (1.2)	30 (7.1)
PaTz-group ^c^	16 (9.8)	83 (19.6)
SCEN physician^d^	83 (50.9)	291 (68.8)
Other	52 (31.9)	57 (13.5)

^a^ *n* = 163 (experience with request or performance in people with dementia). ^b^ *n* = 423. ^c^Group of GPs and district nurses that debate six times a year under the supervision of a palliative care consultant to identify palliative care early, to act proactively. ^d^SCEN: support and consultation on euthanasia in the Netherlands; SCEN physicians are available for support, information, and formal consultation around euthanasia.

### Wishes to increase skills and knowledge related to dementia

Most GPs (*n* = 363; 85.8%) would like to increase their knowledge and skills about dementia care issues (Table 5). GPs especially needed training in legislation and interpretation of the law regarding euthanasia in people with dementia (*n* = 240; 56.7%), and in increasing communication skills to deal with pressure from relatives (*n* = 165; 39%), and knowledge assessing AEDs (*n* = 167; 39.5%).

**Table 5.** Wishes of responders to increase knowledge or skills related to dementia care (*n* = 423)

Training	*n* (%)
Communicating end-of-life aspects	119 (28.1)
Signalling symptoms in cognitive restricted people	137 (32.4)
Dealing with pressure from relatives	165 (39)
Legislation and interpretation of euthanasia regarding people with dementia	240 (56.7)
Advance care planning	98 (23.2)
Disease trajectory of dementia	86 (20.3)
AED	167 (39.5)
No wishes to increase knowledge or skills	60 (14.2)

AED = advanced euthanasia directive.

## Discussion

### Summary

The study quantitatively explored experiences and the subjective burden of euthanasia practice for people with dementia among Dutch GPs. Emotional burden, pressure from relatives and patients, uncertainties, assessment of mental competence, and dealing with AEDs were mentioned as the most burdensome issues. The latter two were significantly more often mentioned when it concerned people with dementia in comparison with other patient groups. The majority of the responders appeared in need of more support than they had used in the past when it concerned a euthanasia request by, or procedure of, a person with dementia. Most often, more support needs from a SCEN physician, a geriatric consultation team, a palliative care consultation team, or the expert centre for euthanasia were mentioned.

### Comparison with existing literature

The large majority of the GPs had at least once performed euthanasia in patients with another disease than dementia, and nearly all had received such requests. About 40% had at least once received a euthanasia request from a person with dementia, while less than 10% had actually performed euthanasia in such a patient. Of the responders who never had had a euthanasia request from a person with dementia, almost half would consider euthanasia when confronted with such a request. This is in line with the rise of euthanasia for people with dementia in the Netherlands and with previous findings.^4,19^ About half of those who would not consider euthanasia for such patients, would always refer such a patient to a colleague or the Dutch expert centre for euthanasia. Indeed, in 2017, of all euthanasia cases, 11.3% were performed by this national expert centre for euthanasia; however, when it concerned people with dementia, this figure was 44.4%.^20^ As just a small number of physicians at the expert centre for euthanasia handle all these euthanasia requests from people with dementia, each of them has a high caseload. The emotional impact on this group of physicians is unknown and needs further exploration.

About half of the GPs estimated to receive and discuss one or more AEDs per month, in which often euthanasia in cases of future dementia is described. One in four GPs in the study felt uncertainty about dealing with AEDs, and even more GPs wanted training to increase their knowledge around AEDs. As an AED can replace an actual, oral euthanasia confirmation in case a person is no longer capable to decide on this, either owing to cognitive impairment, emotional or behavioural problems, carefully discussing and regularly updating it is extremely important.

As advance care planning (ACP) in people with dementia should start early in the disease trajectory,^21–23^ it is recommended that GPs use the moment that a person with dementia shares or wants to discuss an AED to also start ACP. This advance care directive talk should not only discuss end-of-life preferences, but also be based on the person's values and norms, non-medical issues that concern their quality of life, and care preferences.^24^ When such consultations are used to also provide realistic information about the dementia trajectory and its consequences, unrealistic fear for future suffering might be relieved.^25^ This needs further exploration in prospective, controlled studies.^26,27^

The high percentages of GPs that experienced emotional burden and pressure from patients or relatives concerning euthanasia or euthanasia requests by people with dementia, confirms quantitatively recent qualitative studies on this topic.^6,28^ The high percentages of GPs that would like to be supported by a SCEN physician, a geriatric consultation team, a palliative care expert, or the expert centre for euthanasia, is in accordance with the recommendations resulting from two nominal group meetings with all kind of experts in this field.^18^ Strikingly, moral deliberation or spiritual counselling was hardly mentioned by the responders. Perhaps, GPs mistakenly may associate those kinds of support with religion.^29^ Moreover, in primary care such forms of support are hardly available.^30^ To increase attention for, and provision of, spiritual care for older and palliative patients in primary care, the Dutch Ministry of Health Affairs currently invests 7.5 million Euros per year in this domain.^31^ This might increase the awareness of GPs for this kind of support.

The fact that GPs expressed a need of more support from a SCEN physician than they had experienced in the past is remarkable, as in each euthanasia procedure consulting a SCEN physician is obliged. Besides the formal consultation and assessment of the due medical criteria, these physicians can give expert information and advice about legal, ethical, and communicative aspects, as well as emotional support around the procedure.^32^ Apparently, not all GPs experience such support.

### Strengths and limitations

This quantitative study is unique in focusing on the burden and support needs of GPs when confronted with a euthanasia request or trajectory of a person with dementia. The study had a relatively high response rate from all regions of the Netherlands. The relatively high response rate emphasises GPs’ high involvement in this topic,^19^ as other surveys among Dutch GPs mostly had much lower response rates (29.6–41.0%).^33–35^ Responders were representative of the Dutch GP practice with regard to age and sex,^36^ and came from all Dutch regions.

A limitation is the fact that a validated questionnaire was not used, as this did not exist. However, the concept questionnaire was based on two previous studies and a literature review, and was adapted after having received feedback from six experts.

### Implications for practice

It was found that many Dutch GPs experience emotional burden, uncertainty on assessment of patients’ mental competence, handling AEDs, and pressure from relatives and patients concerning euthanasia requests from people with dementia. In line with this, GPs look for more support from other healthcare professionals and training to improve their knowledge and skills on this complex topic. Together with the rise in number and complexity of this caseload, this warrants primary care support and training for the quickly growing end-of-life care needs of patients with dementia and their caregivers.
